# Computational Modeling of Cardiovascular‐Induced Chest Vibrations: A Review and Practical Guide for Seismocardiography Simulation

**DOI:** 10.1002/cnm.70047

**Published:** 2025-05-19

**Authors:** Mohammadali Monfared, Peshala T. Gamage, Ali Loghmani, Amirtahà Taebi

**Affiliations:** ^1^ Department of Bioengineering Lehigh University Bethlehem Pennsylvania USA; ^2^ Biomedical Engineering Program, Mississippi State University Mississippi State Mississippi USA; ^3^ Department of Biomedical Engineering and Science Florida Institute of Technology Melbourne Florida USA; ^4^ Department of Mechanical Engineering Isfahan University of Technology Esfahan Isfahan Iran

**Keywords:** computational modeling, finite element modeling, heart vibrations, seismocardiography

## Abstract

This paper presents a comprehensive examination of finite element modeling (FEM) approaches for seismocardiography (SCG), a non‐invasive method for assessing cardiac function through chest surface vibrations. The paper provides a comparative analysis of existing FEM approaches, exploring the strengths and challenges of various modeling choices in the literature. Additionally, we introduce a sample framework for developing FEM models of SCG, detailing key methodologies from governing equations and meshing techniques to boundary conditions and material property selection. This framework serves as a guide for researchers aiming to create accurate models of SCG signal propagation and offers insights into capturing complex cardiac mechanics and their transmission to the chest surface. By consolidating the current methodologies, this paper aims to establish a reference point for advancing FEM‐based SCG modeling, ultimately improving our understanding of SCG waveforms and enhancing their reliability and applicability in cardiovascular health assessment.

## Graphical abstract

This review presents the current state‐of‐the‐art in computational modeling of cardiovascular‐induced chest vibrations and offers a practical guide for developing such models.

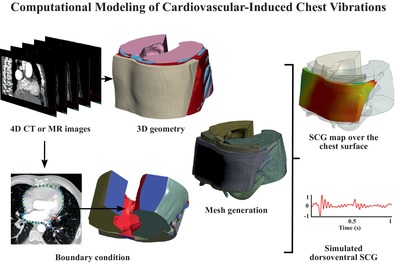

## 1 Introduction

The 2013–2018 National Health and Nutrition Examination Survey and the 2020 U.S. Census predict an increase in the prevalence of cardiovascular diseases (CVDs) for the years 2025–2060. For example, during this period, the projected prevalence of ischemic heart disease, heart failure, myocardial infarction, and stroke combined will increase by 17.4 million cases [1]. This emphasizes the importance of developing more robust and accurate cardiac monitoring methods.

Cardiac monitoring can be done using both invasive and noninvasive methods. The invasive techniques are often performed in clinical facilities and are usually expensive and more complex. On the other hand, noninvasive approaches, such as electrocardiography (ECG), enable remote cardiac monitoring outside of clinical settings, making them more accessible [2, 3, 4]. While ECG provides information about the electrical activity of the heart [5], seismocardiography (SCG), another noninvasive method, complements this by opening a window to the mechanical aspects of heart function [6] by measuring cardiovascular‐induced chest vibrations via placing an accelerometer on the chest. Additional noninvasive imaging options, such as echocardiography and cardiac magnetic resonance imaging (MRI), offer detailed visualizations of cardiac structures and function using ultrasound waves and high‐resolution anatomical data, respectively. These imaging modalities are often considered gold standards but require specialized equipment and clinical environments. On the other hand, ECG‐ and SCG‐based devices stand out for their portability, cost‐effectiveness, and suitability for continuous or at‐home monitoring. Overall, the noninvasive nature of these methods enhances patient comfort and safety compared to invasive procedures [7].

**FIGURE 1:**
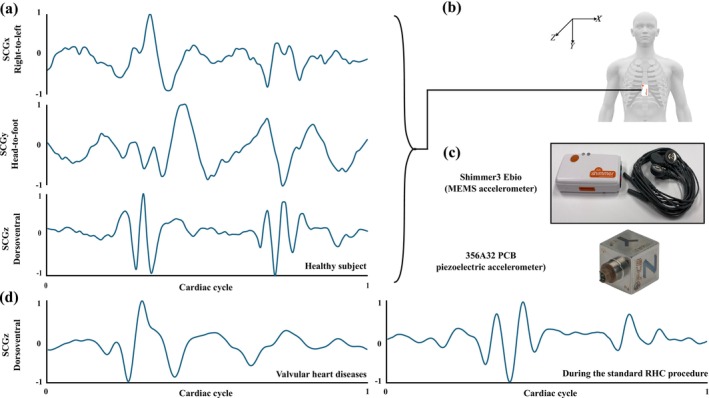
(a) SCG signals of a healthy subject in 3 directions: *X*, *Y*, and *Z* are in the right to left, head‐to‐foot, and dorsoventral directions, respectively [3, 4]. (b) Placement of the sensor on the chest to measure triaxial SCG. The sensor shown in the figure can also measure ECG signals simultaneously. (c) Two types of accelerometers used in SCG measurement: Shimmer3 Ebio (ShimmerSensing, Ireland) and 356A32 (PCB Piezotronics, Depew, NY). (d) Dorsoventral SCG signals of a subject with valvular heart disease [10] and another subject during standard right heart catheterization (RHC) procedure [11] during one cardiac cycle (an ECG P–P interval).

SCG involves detecting vibrations on the chest surface, predominantly originating from the mechanical actions of the heart, including myocardial movements, valve opening and closure, and alterations in blood momentum [7, 8, 9]. Figure 1a shows a sample SCG signal recorded from a healthy adult subject in right‐to‐left (*x*), head‐to‐foot (*y*), and dorsoventral (*z*) directions. SCG has shown promise for diagnosing and monitoring various cardiac conditions and providing complementary information to ECG and other well‐established cardiac monitoring methods [8]. It can be used for continuous monitoring of cardiac activity, which is important for patients with chronic heart conditions [7, 8]. Continuous data collection helps in the early detection of abnormalities and timely intervention [12]. For instance, pulmonary artery pressure is a strong predictor of heart failure outcomes, particularly in patients with existing heart failure. Determining this key indicator of cardiac health in heart failure patients remains challenging because the only FDA‐approved implantable sensor, CardioMEMS, is costly and invasive implantation has limited its use to less than 2% of hospitalized patients [13]. Recently, SCG‐based technologies, have shown promises to overcome these limitations. The SEISMIC‐HF I study showed that the pulmonary capillary wedge pressure (PCWP) can be estimated noninvasively using CardioTag, a wearable device that collects ECG, SCG, and photoplethysmography signals [14]. In this prospective, multisite study of 1000 patients undergoing right heart catheterization, interim results from the first 500 subjects were used to train a machine learning algorithm, demonstrating its potential to accurately identify elevated PCWP (>18 mm Hg) without the need for invasive monitoring. Other studies showed how important cardiac metrics such as preload and stroke volume affect SCG signals in patients with various cardiac diseases [15, 16]. For example, monitoring stroke volume in cardiovascular patients is challenging, as current clinical methods like thermodilution are invasive. Ganti et al. [16] used a chest‐worn wearable sensor to record ECG and SCG signals from 45 patients with congenital heart diseases before and after cardiovascular MRI and applied ridge regression to estimate stroke volume using features such as systolic time. Their model achieved a 72% correlation to the gold‐standard MRI, suggesting the potential of SCG‐based methods in non‐invasively estimating important cardiac health parameters.

To acquire SCG signals, various types of accelerometers, including piezoelectric, capacitive, MEMS, and optical types, can be used to convert cardiovascular‐induced chest vibrations into electrical signals. Proper placement of these sensors on the chest surface is an important factor for reliable SCG signal acquisition. Figure 1b shows a MEMS accelerometer attached to the chest to measure heart‐induced chest vibrations, and Figure 1c represents two types of these accelerometers. Figure 1d shows dorsoventral SCG signals of a subject with valvular heart disease [10] and another subject during the standard right heart catheterization procedure [11] during one cardiac cycle (an ECG P–P interval).

In general, the motion of the chest is influenced by three primary factors: the pumping action of the four chambers of the heart through the main veins and arteries, the impact of breathing, and any extra movement resulting from voluntary or involuntary actions of the individual. The heart is responsible for pumping blood to other organs, and during this process, its walls undergo various movements including anterior–posterior translation and rotation, superior–inferior translation and rotation, and right–left translation and rotation [17]. These movements are transmitted to the surrounding organs and dampened onto the chest surface, where they manifest as visible vibrations [18]. These SCG signals carry information that correlates with both physiological and pathological cardiac activities [19, 20]. However, decoding the morphology of SCG signals and their correlation with their pathophysiological sources presents a challenge due to the complexity of heart movements, coupled with vibrations caused by blood circulation and valve operations. Each of these movements affects the SCG signal, and this highlights the importance of investigating and understanding the origin of SCG waveforms.

**FIGURE 2:**
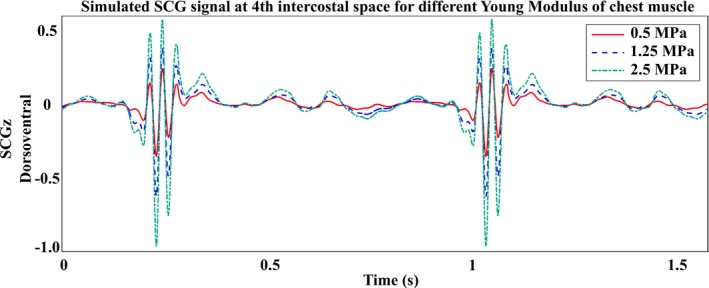
The effect of chest soft tissue stiffness on the dorsoventral SCG waveform modeled at the 4th intercostal space [24].

In this context, computational modeling of cardiovascular‐induced chest vibrations can help in understanding and decoding the SCG waveforms. Finite element modeling (FEM) enables us to create a digital twin of the patient's thorax and conduct sensitivity analysis to study the impact of different parameters, such as anatomical variations, cardiovascular conditions, aging, and physical activities, on SCG waveforms. For instance, by changing the soft tissue thickness on the chest surface, FEM can shed light on the effects of obesity on SCG signals [21]. Furthermore, age‐related changes in muscle properties influence chest muscle stiffness. As muscles age, they generally lose elasticity, leading to increased stiffness [22, 23]. By appropriately modeling the muscles' elasticity in those digital twins, the impact of aging on SCG signals can be evaluated. For instance, Figure 2 shows SCG signals modeled at the 4th intercostal space with different Young's Moduli for the chest soft tissue [24]. In this context, the digital twin models can continuously evolve with the individual's physiological state to improve the accuracy of predictions of the anatomical and physiological variations on SCG signals and potentially guide individualized healthcare recommendations. In this review, we explore the current state‐of‐the‐art for computational modeling of SCG signals.

Computational modeling of SCG signals using FEM involves simulating the vibrations and mechanical activities of the heart as they propagate to the chest surface. These models are instrumental in understanding the genesis of SCG signals, enabling the simulation of various cardiac conditions and their impacts on the SCG waveforms. By replicating the mechanical behavior of the heart and the chest wall, FEM predicts how cardiac motions translate into surface vibrations. In this context, FEM discretizes the structural domain into meshes of finite dimensions, with displacements and forces acting on each element governed by the equations of motion or the wave equation [5, 18, 25].

FEM of cardiovascular‐generated vibrations has been conducted using both 2D and 3D representations. While 2D modeling is more computationally efficient and can provide a simplified representation of cardiac vibration propagation to the chest, 3D models offer a more detailed and accurate picture of these vibrations and the cardiac function. For instance, Gamage et al. [18] employed a 2D computational model that utilized cardiac wall motion extracted from medical images as inlet boundary conditions, successfully replicating features of SCG waveforms and prompting future exploration into 3D modeling using ECG‐gated cardiac magnetic resonance (MR) or computed tomography (CT) scans. Although 2D models may oversimplify the intricate three‐dimensional dynamics of cardiac motion, 3D modeling captures the interactions and spatial motions between organs, yielding critical insights for clinical applications.

**FIGURE 3:**
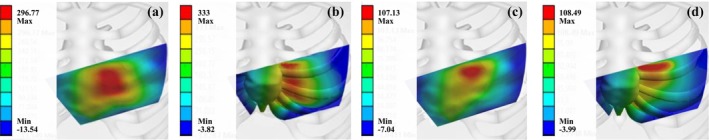
(a) and (c) Modeled SCG distributions on the chest surface. (b) and (d) Accelerations beneath the chest muscle within the thoracic cavity [24].

Gamage [24] further employed medical images and FEM simulations to model the patient‐specific 3D distribution of myocardial motions to the chest surface, establishing a connection between cardiac motions and SCG signals. For example, they investigated how variations in the stiffness of soft tissues, including skin, fat, and muscles, affect the modeled SCG signal output. Their findings revealed that increasing the Young's modulus resulted in a higher amplitude of SCG signal peaks, while the timing of these peaks remained unaffected. These 3D computational models provide a high‐resolution map of chest vibrations caused by cardiac wall motion (Figure 3), offering significantly more detailed information compared to single‐location SCG measurements. Figure 3a,c show the SCG distribution on the chest surface, and Figure 3b,d show the accelerations under the chest muscle within the thoracic cavity [24]. Figure 3a,b are the SCG contours at the time of the first heart sound, and Figure 3c,d are the SCG contours at the time of the second heart sound. These simulated vibrations suggest the power of FEMs in predicting the propagation of cardiac wall motion from the thoracic cavity to the chest surface.

Akhbardeh et al. [5] explored SCG and its relationship with cardiac events using an electromechanical FEM based on diffusion tensor MR data of a canine subject. This approach enabled identification of key cardiac events such as the opening and closure of aortic and mitral valves. They compared and validated the FEM results with the actual SCG signals recorded by accelerometers from human subjects. For this purpose, they attached accelerometers to the subject's sternum and evaluated the SCG fiducial points using the simultaneously recorded echocardiograms. In addition, they derived SCG signals from cine‐MRI data, showing agreement in the timing and shape of the modeled and image‐based acceleration signals, except during the rapid filling phase, which was not clearly captured by cine‐MRI. The comparison of left and right ventricular volume changes with simulated accelerations supported the consistency between modeled dynamics and imaging‐based observations. Their findings indicated that FEM accurately simulated key cardiac events, such as isovolumic contraction and relaxation periods. A later study by Laurin et al. [25] created a physiologically accurate in silico 3D mechanical model of the thorax to investigate its capability in reproducing SCG‐like outputs. Their model incorporated detailed thoracic components such as ribs, costal cartilage, and the xiphoid process to simulate SCG signals, resulting in outputs that exhibited fiducial point analogs, including the mitral valve closure, aortic valve opening, and isovolumic moment points. Furthermore, the simulated SCG displayed oscillatory behavior at the first resonance frequency of the thorax, suggesting that in vivo fiducial points may arise from sudden heart movements followed by regular damped oscillations.

FEM also allows for investigating the impact of various parameters, including anatomical variations, material properties, and blood flow alterations on SCG waveforms. For instance, Sandler et al. [21] assessed how increased soft tissue thickness affects SCG signals, revealing that a 1‐cm increase in chest soft tissue thickness decreased SCG vibration amplitude by factors of 2 and 3 during the first and second heart sounds, respectively.

Despite the utility of FEMs in interpreting SCG features in the context of CVDs, few studies have developed simplified numerical models for SCG signals under such conditions, highlighting a notable gap in the literature. Mithani et al. [26] created a simplified 3D model of an infant heart with single ventricle disease to investigate the relationship between this defect and SCG data. While the material properties in their simulations were not detailed (only stated as using silicon), they concluded that these properties should be adjusted to reflect a lower Young's modulus for more accurate modeling of left ventricular deformation. However, the reliance on data from a single subject may limit the generalizability of their findings, as the output acceleration from a constant hydrostatic pressure on the heart wall only produced one peak, failing to capture the fiducial points observed in the subject's SCG graph. Although Mithani et al. developed a simple pipeline for modeling the dynamics of a heart affected by single ventricle disease, they acknowledged the need for further modifications, including utilizing materials akin to the myocardium and adjusting hydrostatic forces to dynamically represent volume changes during a cardiac cycle. Similarly, Gurev et al. [27] developed a 3D finite element electromechanical canine heart model to simulate SCG signals. The model successfully reproduced major SCG peaks and suggested that these signals reflect the heart's pressure against the ribs. Results also indicated that SCG peaks associated with aortic valve events and blood acceleration stemmed from ventricular contraction and changes in ventricular dimensions during blood ejection. These simulated SCG signals aligned with experimental findings from human volunteers, establishing a correlation between the SCG peak and the maximum acceleration of blood in the aorta, while the first SCG peak following the ECG R‐peak corresponded to aortic valve opening. Collectively, these studies represent pioneering efforts in modeling SCG under pathological conditions, underscoring the need for further research to enhance their accuracy and applicability.

**FIGURE 4:**
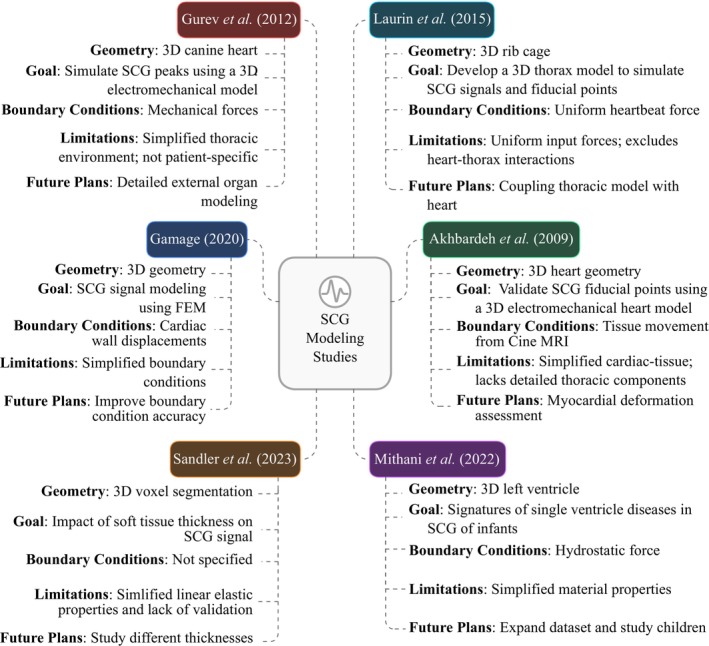
An overview of the current SCG modeling studies.

In conclusion, while the current studies on computational modeling of SCG signals using FEM are among the first in this field, there is considerable room for improvement in the existing approaches. These studies demonstrate the FEM's ability to replicate SCG waveforms and shed light on the relationship between cardiac dynamics and surface vibrations, offering valuable insights for diagnostic applications. However, challenges such as the oversimplification of complex cardiac interactions in 2D models and the reliance on limited subject datasets may hinder the broader applicability of their findings. Figure 4 provides an overview of the studies utilizing FEM for SCG modeling, highlighting their goals, methodologies, and key limitations.

## 2 Governing Equations for SCG Modeling

The mathematical modeling of SCG signals involves the quantification and analysis of mechanical vibrations generated by the cardiovascular system, including the heart, and transmitted through the thoracic cavity to the chest surface. This approach applies principles of biomechanics to characterize the interaction of cardiac motion with adjacent tissues. Central to SCG modeling are the governing equations, such as the equations of motion and the wave equations, which provide a framework for understanding the underlying mechanical dynamics. The equations of motion express the internal forces and movements within the myocardium, capturing the cyclical contraction and relaxation of cardiac muscles, hemodynamic blood flow patterns, and the interactions between the heart and thoracic structures. On the other hand, the wave equations, though not yet applied in SCG‐related studies, offer a promising approach for modeling the propagation of mechanical waves induced by cardiac activity through the chest wall, accounting for the biomechanical properties of tissues such as density and elasticity. It should be noted that the two approaches mentioned here are often considered independently when modeling vibration systems. The application of these mathematical techniques in SCG modeling facilitates the characterization of cardiac mechanics and contributes to a better understanding of SCG waveforms.

### 2.1 Equations of Motion

Modeling SCG signals using the equations of motion involves analyzing the mechanical behavior of the chest wall as it responds to the heartbeat. The equations of motion, derived from Newton's second law, provide a framework to describe how forces affect the motion of an object. In modeling SCG, this approach involves setting up differential equations that account for the forces exerted by the heartbeats, the damping due to tissue properties, and the stiffness of the chest wall. In this context, modeling SCG can be framed as a forced vibration problem, where the system oscillates in response to an external variable force or displacement, with the heart serving as the main source of chest vibrations and the cardiac wall motion inducing vibrations in the surrounding organs. For instance, Gamage et al. [18] utilized this approach for 2D modeling of the SCG signals by simulating the distribution of heart wall motion to the chest surface. Mithani et al. [26] also used this method to establish the correlation between single ventricle disease and SCG signals in neonatal heart models. The equations of motion can be expressed in matrix form as:

(1) $$\left\{f\left(t\right)\right\}=\left[M\right]\left\{\ddot{q}\right\}+\left[C\right]\left\{\dot{q}\right\}+\left[K\right]\left(\left\{q\right\}-\left\{u\right\}\right)$$

where $\left\{u\right\}$ is a prescribed or reference displacement vector due to boundary conditions. This term introduces relative motion into the system, e.g., due to the heart motion, affecting the response through the stiffness matrix. In (1), $f\left(t\right)$ represents the vector of applied forces, $\left[M\right]$ is the mass matrix, $\left[C\right]$ is the damping matrix, $\left[K\right]$ is the stiffness matrix, $\left\{\ddot{q}\right\}$ is the acceleration vector, $\left\{\dot{q}\right\}$ is the velocity vector, and $\left\{q\right\}$ is the displacement vector.

In time‐domain analysis under the assumption of a linear time‐invariant system, while system properties such as stiffness and damping are not time‐dependent, the displacement $\left\{q\right\}$, velocity $\left\{\dot{q}\right\}$, acceleration $\left\{\ddot{q}\right\}$, and force $\left\{f\right\}$ vectors all vary with time, making the analysis time‐dependent. This is a common approach when modeling biological tissues, as their mechanical properties, such as stiffness and damping, are generally considered constant during the short time frames of cardiac cycles. However, if capturing more complex behaviors, such as tissue viscoelasticity or dynamic changes in tissue properties over time (e.g., due to heart rate variability, breathing, or pathological changes), is needed, the stiffness $\left[K\right]$ or damping $\left[C\right]$ can also be considered time‐dependent. In that context, different types of damping can be considered while solving (1):

1. Constant damping.
2. Material damping, which is defined in the material definition. This type of damping represents internal friction within the material due to molecular interactions.
3. Rayleigh damping, which assumes that the damping matrix $\left[C\right]$ is linearly proportional to the mass and stiffness matrices as $\left[C\right]=\alpha \left[M\right]+\beta \left[K\right]$, where α and β are the mass and stiffness multiplier terms, respectively. Rayleigh damping is suitable for dynamic analyses where both mass and stiffness play a role in energy dissipation. It is important to note that while Rayleigh damping can be used as a simplified approximation for modeling heart vibrations, particularly when balancing computational efficiency and capturing basic damping effects, it may not fully represent the complex, nonlinear, and frequency‐dependent behavior of biological tissues. To calculate the acceleration on the chest surface, assuming a Rayleigh damping, (1) can be written as follows:

(2) $$\left\{f\left(t\right)\right\}=\left[M\right]\left\{\ddot{q}\right\}+\left(\alpha \left[M\right]+\beta \left[K\right]\right)\left\{\dot{q}\right\}+\left[K\right]\left(\left\{q\right\}-\left\{u\right\}\right)$$

### 2.2 Damped Elastic Wave Propagation Equation

The linear wave equation provides a framework for describing the evolution of these vibrations over time and space. While equations of motion elucidate the forces acting among thoracic organs, the damped elastic wave propagation equation effectively captures the mechanics of how heart vibrations are transmitted through various media, including soft tissues and bone structures. The wave characteristics are influenced by the biomechanical properties of the surrounding tissues, such as density, elasticity, and intrinsic damping characteristics. For example, the vibrations can manifest as longitudinal waves (where particle displacement aligns with wave direction) or transverse waves (where particle displacement is perpendicular), with their propagation speed determined by the material properties of the chest and surrounding tissues. Accurately modeling damping mechanisms, such as viscoelastic damping, is critical in biological materials because they account for energy dissipation and the amplitude attenuation of the wave as vibrations travel from the heart to the chest surface. Additionally, the boundaries between different tissues, such as those of the thoracic cavity, can result in reflections and refractions that further influence wave behavior [28]. The wave equation for a homogeneous and isotropic medium can be expressed as follows:

(3) $$\frac{\partial^{2}q}{\partial t^{2}}=c^{2}\nabla^{2}q$$

where q is the displacement field, c is the speed of wave propagation in the medium, ∇ is the Nabla operator ($=\partial^{2}/\partial x_{1}^{2}+\partial^{2}/\partial x_{2}^{2}+\partial^{2}/\partial x_{3}^{2}$), and t is the time. This equation can be utilized, with appropriate boundary and initial conditions reflective of physiological scenarios, to simulate how cardiac‐induced vibrations propagate through the chest wall [29]. While the wave equation assumes a perfectly elastic medium, realistic modeling of heart vibrations necessitates incorporating damping effects characteristic of biological tissues. The damped elastic wave propagation equation can be formulated as follows:

(4) $$\rho \left(x\right)\frac{\partial^{2}q}{\partial t^{2}}=\nabla \cdot \left(C\left(x\right)\epsilon \left(q\right)\right)+\nabla \cdot \left(c_{k}\left(x\right)C\left(x\right)\epsilon \left(\frac{\partial q}{\partial t}\right)\right)$$

where:

$x=\left(x_{1},x_{2},x_{3}\right)$: 3D position vector.

$q\left(x,t\right)$: Displacement vector at position x and time t.

$\rho \left(x\right)$: Density of the material.

$c_{k}\left(x\right)$: Stiffness‐proportional damping coefficient.

$C\left(x\right)$: Elasticity tensor.

$\epsilon \left(q\right)$: Strain tensor, defined as:

$$\epsilon \left(q\right)=\frac{1}{2}\left(\nabla q+{\left(\nabla q\right)}^{T}\right).$$

In (4), the first term on the right‐hand side, $\nabla \cdot \left(C\left(x\right)\epsilon \left(q\right)\right)$, accounts for the elastic response of the tissue, while the second term introduces the damping effect, proportional to the strain rate. This incorporation of damping directly influences how heart vibrations are transmitted and perceived at the chest surface, enhancing the realism of the model. To investigate the mechanical transmission of thoracic vibrations from the heart, Laurin et al. [25] utilized the conventional equations for damped elastic wave propagation. This approach highlights the potential utility of the damped elastic wave propagation equation in modeling the complex dynamics of cardiac vibrations as they propagate through the chest.

## 3 Challenges and Approaches in Defining Boundary Conditions

Boundary conditions are crucial in FEM of heart vibrations, as they define how the model interacts with its environment and ensure that simulations accurately replicate physiological behaviors. These conditions are vital for capturing the mechanical dynamics of the heart and translating them into surface vibrations detectable by SCG. However, the implementation of boundary conditions presents several challenges; inaccuracies in defining these parameters can lead to significant modeling errors, potentially misrepresenting the heart's vibrational characteristics and undermining the reliability of SCG simulations.

To simulate the chest vibrations generated during the heart's pumping cycle using FEM, various approaches have been utilized for boundary conditions in the literature. A common method involves employing patient‐specific displacements of the heart wall, typically derived from imaging modalities such as 4D cardiac MRI or CT scans, as the input boundary condition [5, 18, 24]. This approach relies on image processing and motion tracking techniques, where the accuracy of cardiac motion tracking directly impacts the fidelity of the resultant vibrational data. Precise mapping of these boundary conditions is crucial for accurately capturing the vibrational patterns associated with cardiac activity, as any discrepancies can lead to a flawed representation of heart‐induced vibrations.

Alternatively, some studies adopt a more generalized approach, applying force functions to represent the heartbeat [25] or exerting various forces on simplified geometries to approximate deformations similar to those of the heart wall [26]. While these methodologies can simplify the computational process, they often require a trial‐and‐error approach to fine‐tune parameters related to cardiac vibrations, which may not consistently yield reliable and patient‐specific outcomes. This reliance on approximation highlights the importance of balancing model complexity with computational efficiency, as oversimplification can obscure essential physiological details critical for interpreting SCG signals.

## 4 Meshing Strategies and Considerations

Meshing allows for the discretization of the computational domain and enables accurate predictions of structural responses to loads and boundary conditions associated with heart vibrations. FEM breaks down complex geometries of the thoracic organs and tissues into smaller, manageable parts known as mesh elements. These elements can vary in shape and order, including linear and quadratic forms, tetrahedral and hexahedral configurations, as well as specialized forms like shell elements [26], 2D elements [18], and 3D solid elements [24, 30]. The choice of meshing strategy is critical, particularly when modeling the mechanical behavior of the heart and its resultant vibrations. Representing a geometry as a shell assumes a predominantly two‐dimensional behavior, where the thickness is negligible compared to the length and width. This approach may be suitable for certain aspects of cardiac mechanics; however, it can limit the accurate representation of vibrational dynamics that are inherently three‐dimensional. Conversely, modeling the heart as a 3D solid captures the full complexity of its mechanical behavior, including the intricate interactions and spatial variations of the cardiac structures [31, 32, 33]. The choice between these meshing strategies ultimately affects the fidelity of SCG simulations, as it influences the accuracy of the predicted vibrations at the chest surface.

## 5 Material Models in SCG Simulation

In SCG modeling, understanding and incorporating realistic material properties of the human thorax is important to get an accurate output signal. The material properties affect how mechanical waves propagate through the body. Table 1 lists the material properties utilized in the SCG modeling literature. Gamage et al. [18, 30] utilized both linear elastic and hyperelastic material models in SCG simulations. Linear elastic models, commonly employed for soft tissues due to their simplicity and computational efficiency, are defined mathematically by Hooke's Law, which relates stress to strain in elastic materials. In three‐dimensional space, this relationship is described by the generalized form of Hooke's Law for isotropic materials [49], expressed as $\sigma_{\mathrm{ij}}=C_{\text{ijkl}}\epsilon_{\mathrm{kl}}$, where $\sigma_{\mathrm{ij}}$ is the stress tensor, $C_{\text{ijkl}}$ is the fourth‐rank stiffness tensor, and $\epsilon_{\mathrm{kl}}$ represents the strain tensor. For isotropic materials, the stiffness tensor simplifies using Young's modulus (E) and Poisson's ratio (ν), yielding the stress–strain relationship: $\sigma_{\mathrm{ij}}={\lambda \delta }_{\mathrm{ij}}\epsilon_{\mathrm{kk}}+2\mu \epsilon_{\mathrm{ij}}$. Here, λ and μ are the Lamé constants, which are related to E and ν by $\lambda =\frac{\mathrm{E\nu}}{\left(1+\nu \right)\left(1-2\nu \right)}$ and $\mu =\frac{E}{2\left(1+\nu \right)}$, respectively. The term $\delta_{\mathrm{ij}}$ is the Kronecker delta, while $\epsilon_{\mathrm{kk}}$ denotes the trace of the strain tensor, i.e., the sum of the normal strains. The strain tensor $\epsilon_{\mathrm{ij}}$ is related to the displacement field $q_{i}$ through the equation $\epsilon_{\mathrm{ij}}=\frac{1}{2}\left(\frac{\partial q_{i}}{\partial x_{j}}+\frac{\partial q_{j}}{\partial x_{i}}\right)$. The stress components in the stress tensor $\sigma_{\mathrm{ij}}$ can be explicitly written as $\sigma_{\mathrm{xx}}=\lambda \left(\epsilon_{\mathrm{xx}}+\epsilon_{\mathrm{yy}}+\epsilon_{\mathrm{zz}}\right)+2\mu \epsilon_{\mathrm{xx}}$, $\sigma_{\mathrm{yy}}=\lambda \left(\epsilon_{\mathrm{xx}}+\epsilon_{\mathrm{yy}}+\epsilon_{\mathrm{zz}}\right)+2\mu \epsilon_{\mathrm{yy}}$, $\sigma_{\mathrm{zz}}=\lambda \left(\epsilon_{\mathrm{xx}}+\epsilon_{\mathrm{yy}}+\epsilon_{\mathrm{zz}}\right)+2\mu \epsilon_{\mathrm{zz}}$, $\sigma_{\mathrm{xy}}=2\mu \epsilon_{\mathrm{xy}}$, $\sigma_{\mathrm{xz}}=2\mu \epsilon_{\mathrm{xz}}$, and $\sigma_{\mathrm{yz}}=2\mu \epsilon_{\mathrm{yz}}$.

**TABLE 1:** Material models and properties utilized in SCG modeling studies.

Paper	Thoracic component	Material model	Young's modulus	Density	Poisson ratio	Damping parameter
Gamage et al. [18]	Lungs and surrounding tissues	Linear elastic	—	—	—	—
Gamage [24]	Chest muscle	Linear elastic	0.5–2.5 MPa [34]	1000 kg/m^3^ [35]	0.3 [36]	—
	Sternum, ribs, xiphoid		12 GPa [37]	2000 kg/m^3^ [37]	0.4 [37]	—
	Costal cartilage		3 GPa [34]	2000 kg/m^3^ [34]	0.4 [34]	—
	Intercostal muscle		3 MPa [34]	1000 kg/m^3^ [34]	0.4 [34]	—
	Lungs	Hyperplastic	Mooney–Rivlin 5th order (C10 = −859.78 Pa, C01 = 947.5 Pa, C20 = 1783.2 Pa, C11 = −5440.5 Pa, C02 = 4633.5 Pa) [38]	1250 kg/m^3^ [39]	—	—
Laurin et al. [25]	Cortical bone	Viscoelastic	3.8 GPa [40, 41, 42, 43]	2000 kg/m^3^ [40, 42]	0.3 [40]	0.067 [42]
	Cancellous bone		3.0 GPa [40, 44]	2000 kg/m^3^ [44]	0.3 [40]	0.067 [42]
	Cartilage		5.2 MPa [45]	1500 kg/m^3^ [40, 43]	0.3 [40]	0.13 [42]
	Lungs	Viscoelastic	0.1 MPa [46]	280 kg/m^3^ [46, 47]	0.4 [46]	—
Mithani et al. [26]	Myocardium [48]	Linear elastic	3.05 MPa [48]	—	—	—

Unlike linear elastic models, a viscoelastic model encompasses both elastic and viscous properties, indicating that the material's response to stress involves time‐dependent deformation. While muscles exhibit viscoelastic behavior, they can often be effectively modeled as linear elastic materials under certain assumptions [50]. These assumptions include operating over short time scales where viscoelastic effects are negligible, considering small strains, assuming isotropic material properties, and neglecting factors such as muscle activation and contraction [51]. In simulations of cardiac motion propagation to the chest surface, the relevant time scale is extremely brief (corresponding to a single cardiac cycle) during which the chest muscles remain in a passive state. These simplifications facilitate a more manageable analysis of the complex behavior of muscles. However, when needed, to model the viscoelastic behavior of materials, various mathematical frameworks exist, including the Maxwell model, the Kelvin–Voigt model, and the standard linear solid model [52, 53].

The Maxwell model, which consists of a spring representing the elastic element and a dashpot representing the viscous element arranged in series, captures the time‐dependent response of materials under stress. Its constitutive equation is given by $\sigma \left(t\right)=E\epsilon \left(t\right)+\eta \frac{d\epsilon \left(t\right)}{\mathrm{dt}}$, where $\sigma \left(t\right)$ represents the stress at time t, E is the Young's modulus of the spring, $\epsilon \left(t\right)$ is the strain at time t, η denotes the viscosity of the dashpot, and $\frac{d\epsilon \left(t\right)}{\mathrm{dt}}$ is the time derivative of strain. This arrangement allows the material to exhibit both instantaneous elastic behavior and time‐dependent viscous behavior [53].

In contrast, the Kelvin–Voigt model consists of a spring and a dashpot arranged in parallel, which results in a different stress–strain relationship. The constitutive equation for the Kelvin–Voigt model is also $\sigma \left(t\right)=E\epsilon \left(t\right)+\eta \frac{d\epsilon \left(t\right)}{\mathrm{dt}}$ with the same definitions for $\sigma \left(t\right)$, E, $\epsilon \left(t\right)$, η, and $\frac{d\epsilon \left(t\right)}{\mathrm{dt}}$. However, in this configuration, the model captures the material's resistance to both immediate and time‐dependent deformation, highlighting its ability to retain strain without significant relaxation over time. These distinct arrangements and behaviors of the Maxwell and Kelvin–Voigt models are crucial for understanding viscoelastic materials in various applications [52, 54].

The standard linear solid model, also known as the Zener model, combines features from both the Maxwell and Kelvin–Voigt models. Its constitutive equation is expressed as $\sigma \left(t\right)+\tau_{1}\frac{\mathrm{d\sigma}\left(t\right)}{\mathrm{dt}}=E_{1}\epsilon \left(t\right)+\left(E_{2}\tau_{2}\right)\frac{d\epsilon \left(t\right)}{\mathrm{dt}}$, where $\tau_{1}$ and $\tau_{2}$ are relaxation times, and $E_{1}$ and $E_{2}$ are the elastic moduli of the springs [55].

For a more comprehensive understanding of viscoelastic behavior, a general representation employs a convolution integral involving a relaxation function $G\left(t\right)$. The stress–strain relationship can be defined as $\sigma \left(t\right)=\int_{0}^{t}G\left(t-\tau \right)\frac{d\epsilon \left(\tau \right)}{\mathrm{d\tau}}\mathrm{d\tau}$, where $G\left(t\right)$ describes how the material relaxes stress over time, and $\epsilon \left(\tau \right)$ is the strain as a function of past time τ. Additionally, the differential form of a general linear viscoelastic constitutive equation can be expressed as $\sum_{i=0}^{n}a_{i}\frac{d^{i}\sigma \left(t\right)}{{\mathrm{dt}}^{i}}=\sum_{j=0}^{m}b_{j}\frac{d^{j}\epsilon \left(t\right)}{{\mathrm{dt}}^{j}}$, where $a_{i}$ and $b_{j}$ are material constants, and n m represent the orders of the derivatives.

While some research has focused on viscoelastic materials for FEM of the lung [56], the complex, nonlinear, and large deformation behavior of lung tissue often necessitates hyperelastic models. This is critical for accurately analyzing the stress–strain response of lung tissue during breathing, disease progression, or rapid changes in pressure or volume conditions [57]. For example, Maghsoudi‐Ganjeh et al. [58] utilized a compressible Mooney–Rivlin hyperelastic model to simulate lung behavior. The Mooney–Rivlin model is a widely used hyperelastic material model that describes the nonlinear stress–strain behavior of rubber‐like materials [59, 60]. This model is particularly effective for materials that undergo large elastic deformations. The Mooney–Rivlin strain energy density function W is expressed as $W=\sum_{i,j}C_{\mathrm{ij}}{\left({\bar{I}}_{1}-3\right)}^{i}{\left({\bar{I}}_{2}-3\right)}^{j}$, where $C_{\mathrm{ij}}$ are material constants, and ${\bar{I}}_{1}$ and ${\bar{I}}_{2}$ are the first and second invariants of the deviatoric part of the left Cauchy‐Green deformation tensor. For the most common first‐order Mooney–Rivlin model, the strain energy density function simplifies to $W=C_{10}\left({\bar{I}}_{1}-3\right)+C_{01}\left({\bar{I}}_{2}-3\right)$, where $C_{10}$ and $C_{01}$ are material constants. In higher‐order models, additional terms such as ${\left({\bar{I}}_{1}-3\right)}^{2}$, ${\left({\bar{I}}_{2}-3\right)}^{2}$, and $\left({\bar{I}}_{1}-3\right)\left({\bar{I}}_{2}-3\right)$ are included, each associated with its own material constant.

Although hyperelastic models are commonly used to describe the stress–strain relationship in biological soft tissues, such as muscles, they are not ideal for representing time‐dependent processes like stress relaxation. This limitation arises because hyperelastic models are inherently time‐independent, despite being effective for capturing the nonlinear behavior of soft tissues [61]. In contrast, modeling the mechanical behavior of bones and cartilage, such as in the sternum, ribs, and xiphoid process, often is implemented by different approaches. For example, Gamage et al. used a linear elastic model [18, 30], while Laurin et al. [25] applied a viscoelastic model. Within the SCG modeling framework, comparisons of linear elastic and viscoelastic models for chest bones indicate that linear elastic models, though computationally simpler, are often sufficient. However, viscoelastic models, while more complex, are better suited to capturing the time‐dependent behavior of bones under dynamic and long‐term loading. Despite various material choices, there are currently no published studies directly comparing different constitutive models (e.g., hyperelastic vs. viscoelastic) in SCG simulations. Only Gamage [24] (Figure 2) investigated how varying the Young's modulus of chest wall muscles at the 4th intercostal space affects SCG signals. Such comparisons can shed light on the impact of these parameters in accurate SCG modeling using FEM.

## 6 Output SCG Signal

**FIGURE 5:**
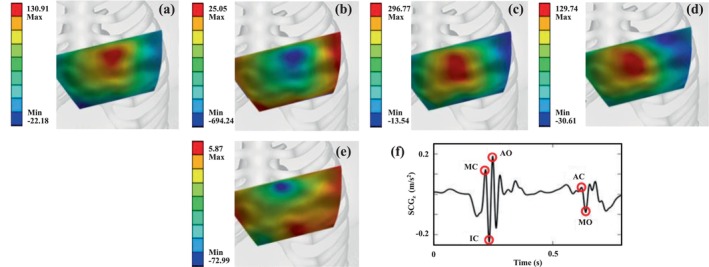
Computationally modeled dorsoventral SCG maps at the timing of key fiducial points. (a) Mitral valve closure, MC. (b) Isovolumic contraction, IC. (c) Aortic valve opening, AO. (d) Aortic valve closure, AC. (e) Mitral valve opening, MO. (f) These fiducial points are labeled on the dorsoventral SCG signal measured at the 5th intercostal space. All measurements are in $\mathrm{mm}/s^{2}$ [24].

SCG signal varies depending on the specific measurement location on the chest surface [6, 8]. In this context, FEM of cardiovascular‐induced chest vibrations offers a high‐resolution, time‐varying map of SCG signals, allowing for the analysis of both temporal and spatial variations. Such an analysis could enhance the current understanding of how SCG signals and the cardiovascular information they provide are influenced by the measurement location on the chest. Moreover, since patient‐specific SCG modeling requires 4D imaging data, such as 4D cardiac CT or MR, simulated SCG signals can be synchronized with these images to identify key cardiac events and intervals, including valve opening and closure, rapid ejection and filling, and isovolumic contraction and relaxation. For example, Figure 5 shows how the chest accelerations vary during a cardiac cycle at specific fiducial points [24]. These computational models can also be adjusted to simulate various cardiac conditions (e.g., by introducing a septal defect or increasing the thickness of the cardiac wall), offering insights into how SCG waveforms and fiducial points change under pathological conditions. Acceleration, displacement, and velocity on the chest surface are key outputs derived from FEM modeling of SCG signals. By calculating, validating, and evaluating these signals, experts can assess patient‐specific results tailored to individual physiological profiles.

## 7 Validation Methods

While several studies have made valuable contributions to modeling SCG signals, comprehensive validation remains limited. Some studies did not report validation of their FEM [21]. In other cases, the modeled SCG signals derived from imaging data, such as CT scans, of one subject were compared to SCG signals recorded by accelerometers from different human subjects, with validation based primarily on general morphological similarities [24, 25, 27]. Additionally, one study evaluated SCG signals generated from an electromechanical model of a canine heart by comparing them to SCG signals estimated from cine‐MRI data of human subjects [5]. In addition to direct validation approaches, several studies have explored complementary strategies that can help assess the physiological plausibility of modeled SCG signals. While these methods do not provide direct validation of the SCG waveform itself, they can confirm the accuracy of physiological information extracted from the models. For example, morphological features and fiducial points identified in simulated SCG signals have been compared with known cardiac events derived from ECG or cardiac imaging [5, 24]. Left ventricular volume curves obtained from 4D CT or MRI have been used to interpret the modeled SCG‐derived parameters such as AO and MC [18]. Similarly, simultaneously recorded ECG signals enable temporal alignment of SCG features with electrical events like the R‐peak, helping to confirm the physiological consistency of the modeled signals [24].

These limitations highlight the need for more rigorous and subject‐specific validation approaches. Validation of computational simulations in SCG modeling is essential to ensure the accuracy and reliability of the modeled SCG signals. Various methods, including accelerometer measurements, advanced motion tracking systems using medical imaging, and laser Doppler vibrometry, can be employed, each with distinct strengths and limitations.

Using accelerometers is a standard method for directly measuring mechanical vibrations or accelerations on the chest surface, which makes it an effective validation tool for SCG modeling. By placing single or arrays of accelerometers on specific locations of the chest, such as the sternum or ribs, SCG signals can be captured in multiple directions (e.g., dorsoventral, right‐to‐left, and head‐to‐foot). These signals serve as a gold standard for validation because they represent real physical responses that can be compared to simulated data. However, this validation approach has limitations. It requires precise sensor placement and careful calibration, which can be logistically challenging. Moreover, capturing SCG signals with uniaxial or triaxial accelerometers may not fully account for all mechanical changes across the chest surface [62]. Sensor arrays also measure SCG signals from limited chest locations, while useful for validating modeled SCG signals at those specific sites, may not provide sufficient data to validate the entire modeled chest vibration map.

Another potential validation approach involves using computer vision‐based SCG to estimate SCG maps from chest videos [63, 64]. This new non‐contact approach may offer a significant advantage over conventional techniques by capturing SCG signals from a much larger number of chest locations. However, a key limitation is its current inability to capture signals in the dorsoventral direction, as it only provides data for right‐to‐left and top‐to‐bottom vibrations. This restriction limits its effectiveness in fully validating SCG maps. Performing either this approach or the accelerometer‐based method concurrently with 4D medical imaging, used for geometry segmentation and boundary condition evaluation, is challenging. One potential solution is to capture validation data immediately before or after the 4D MRI or CT scans, assuming that the SCG signals remain relatively stable within the short time frame between tests. While this assumption is reasonable, it is important to note that the modeled SCG signals derived from the medical images may differ slightly from the validation signals due to inter‐subject variability in SCG beats, meaning that SCG waveforms can vary from one beat to the next.

In addition to these validation methods, computer vision techniques can be used to track chest surface motions derived directly from the same medical imaging data used for extracting geometry and boundary conditions. In this case, the image data such as MR, CT, or ultrasound images serves a dual purpose: providing anatomical details and boundary condition information as well as validation data. To extract validation data from these images, motion‐tracking techniques can be used to monitor the displacement, velocity, and acceleration of specific anatomical points on the chest. For instance, motion‐tracking algorithms could target the xiphoid process or other key regions of interest. The motion profiles of these points can be directly compared with the outputs of the SCG computational model, providing a robust means of assessing model accuracy. One notable advantage of this approach is its ability to generate validation data that is temporally aligned with the modeled SCG signals, facilitating a direct “apples‐to‐apples” comparison. Unlike the computer vision approach based on chest videos, this approach inherently captures chest motion in all spatial directions, including the dorsoventral axis, offering a more comprehensive dataset for validation. However, this method has its own limitations. High‐quality imaging systems necessary for precise motion capture can be cost‐prohibitive and may not be readily available in all research or clinical environments. Additionally, imaging procedures often require significant technical expertise, and patient movement during scanning can introduce noise or artifacts, potentially affecting the accuracy of the extracted motion data. Despite these challenges, this technique holds promise for providing high‐fidelity validation data, particularly in controlled settings where imaging resources are accessible.

## 8 Sample Fem Framework for SCG Simulation

**FIGURE 6:**
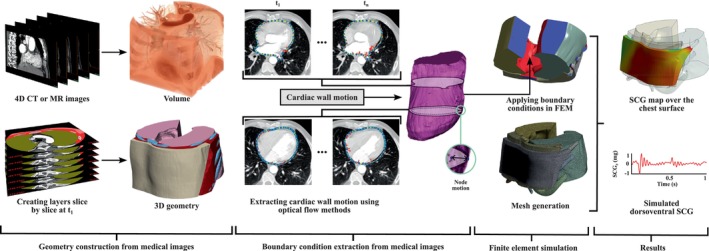
Overview of a sample image‐based patient‐specific FEM of SCG signals. The framework begins with the processing of MRI or CT scan images to reconstruct the 3D geometry of the heart and surrounding thoracic structures. Cardiac wall motion is then extracted using motion tracking algorithms like the Lucas–Kanade method [65, 66, 67]. This motion data is applied as input boundary conditions to the 3D geometry within the FEM solver. The final steps involve mesh generation and setting up the analysis parameters, ultimately allowing for the computation and analysis of chest surface acceleration.

This section provides a sample framework for FEM‐based SCG modeling, offering guidance for future studies aiming to advance the field. Figure 6 outlines this framework, beginning with the acquisition of MRI or CT scan images. Many of the studies referenced earlier employ common techniques such as geometry generation from medical images. Using image processing, the 3D geometry of the heart and surrounding thoracic structures is reconstructed from these scans. To model the propagation of cardiac wall motion to the chest surface, the equations of motion can be employed. This process involves translating known heart wall motions from 4D cardiac CT scans into dynamic boundary conditions, which are then used to simulate the mechanical response of the surrounding thoracic tissues. The methodology consists of several key steps: defining the equations of motion for continuous media, converting these to a discrete matrix form suitable for FEM, and applying boundary conditions derived from 4D CT data. By following this process, the propagation of cardiac vibrations through the thoracic structures to the chest surface can be simulated. In the Subsections 8.1 through 8.5, alongside a detailed discussion of the fundamental concepts, the sample framework illustrated in Figure 6 is explained for readers seeking a deeper understanding of the methodology.

### 8.1 Equations of Motion for Continuous Media

The equations of motion for continuous media are derived from Newton's second law and are expressed in terms of stress, strain, and body forces. The primary form is given by $\rho \frac{\partial^{2}q}{\partial t^{2}}=\nabla \cdot \sigma +b$, where ρ is the density of the material, q is the displacement vector, σ is the stress tensor, b represents body forces (e.g., gravity), and ∇⋅σ denotes the divergence of the stress tensor. The stress tensor σ is related to the strain tensor ϵ through the material's constitutive law. For linear elastic materials, this relationship is described by Hooke's law: $\sigma_{\mathrm{ij}}=C_{\text{ijkl}}\epsilon_{\mathrm{kl}}$, where $C_{\text{ijkl}}$ is the elasticity tensor, and $\epsilon_{\mathrm{kl}}$ is the strain tensor defined as $\epsilon_{\mathrm{kl}}=\frac{1}{2}\left(\frac{\partial q_{k}}{\partial x_{l}}+\frac{\partial q_{l}}{\partial x_{k}}\right)$. For non‐linear materials, hyperelastic models like the Mooney–Rivlin or Ogden models can be used. In the context of finite element analysis, the continuous domain is discretized into finite elements, and the equations of motion are expressed in a matrix form suitable for numerical solution as described by (1). Figure 6 highlights how the reconstructed 3D geometry is used to apply the equations of motion. By discretizing the thoracic structures slice by slice, the FEM framework incorporates the anatomical details necessary for accurate simulation of SCG signal propagation.

### 8.2 Applying Boundary Conditions From 4D CT Data

Displacement boundary conditions are extracted from 4D CT scans and applied to the finite element model. These time‐dependent conditions are specified at the nodes on the heart's surface, reflecting the dynamic motion observed in the CT data: $q\left(x,t\right)=q_{\text{data}}\left(x,t\right)$, where $q_{\text{data}}\left(x,t\right)$ represents the displacement data obtained from the 4D CT scans. These boundary conditions ensure that the model accurately reflects the dynamic behavior of the heart as captured in the imaging data. Figure 6 illustrates the extraction of cardiac wall motion from 4D imaging data using optical flow methods. This extracted motion, shown as displacement fields over time, forms the dynamic boundary conditions applied to the FEM model, ensuring that the heart's motion drives the mechanical response of surrounding tissues.

### 8.3 Finite Element Discretization and Solution

The continuous domain is discretized into finite elements, and the weak form of the equations of motion is derived and solved numerically. The weak form is obtained by multiplying the equations of motion by a test function v and integrating over the domain Ω: $\int_{\Omega }\rho \frac{\partial^{2}q}{\partial t^{2}}\cdot v\;d\Omega =\int_{\Omega }\left(\nabla \cdot \sigma \right)\cdot v\;d\Omega +\int_{\Omega }b\cdot v\;d\Omega$. Using the divergence theorem, this can be rewritten as $\int_{\Omega }\rho \frac{\partial^{2}q}{\partial t^{2}}\cdot v\;d\Omega =\int_{\Omega }\sigma :\nabla v\;d\Omega +\int_{\Omega }b\cdot v\;d\Omega -\int_{\partial \Omega }t\cdot v\;d\Gamma$, where t is the traction vector on the boundary ∂Ω. The weak form provides the foundation for numerically solving the system of equations by discretizing the model into finite elements. The mesh generation step in Figure 6 demonstrates how the reconstructed 3D geometry is discretized into finite elements. This discretization enhances the accuracy of the simulation by ensuring the mechanical response of the tissues is captured with sufficient detail.

### 8.4 Time Integration

For dynamic analysis, time integration schemes like the Newmark‐beta method are used to solve the equations of motion. The Newmark‐beta method updates the displacement and velocity at each time step n+1 based on the current values at time step n: $q_{n+1}=q_{n}+\Delta tv_{n}+\frac{\Delta t^{2}}{2}\left(\left(1-2\beta \right)a_{n}+2\beta a_{n+1}\right)$ and $v_{n+1}=v_{n}+\Delta t\left(\left(1-\gamma \right)a_{n}+\gamma a_{n+1}\right)$, where Δt is the time step, and β and γ are parameters that determine the stability and accuracy of the method. Common choices are β=1/4 and γ=1/2, which correspond to the average acceleration method. This selection ensures that the Newmark‐beta method is unconditionally stable for linear problems, providing second‐order accuracy and sufficient numerical damping to control high‐frequency oscillations. Using medical images and optical flow methods, the cardiac motion data is translated into time‐dependent inputs, which are applied to the heart cavity as boundary conditions. The sampling frequency of these inputs plays a critical role in the accuracy of the results. By increasing the sampling frequency of the input data, the simulation can capture hidden peaks and reveal more detailed patterns in the simulated SCG graph and potentially enhance the analysis of cardiac events.

### 8.5 Solving the Equations

The global system of equations is assembled from the finite element discretization and solved iteratively at each time step using numerical solvers software. The solution involves calculating the displacement, velocity, and acceleration fields throughout the thoracic cavity and on the chest surface. The computational models enable the capture of SCG signals and displacement at any point on the chest surface in three directions, a task that is challenging in experimental settings. FEM also provides the flexibility to modify input boundary conditions, such as those specific to an individual with a particular heart defect, allowing for the study of patient‐specific SCG signals.

## 9 Limitations and Future Work

The field of SCG is relatively young but holds great potential for growth and impact. In recent years, SCG signals have garnered increasing attention, especially with the promising results from the SEISMIC‐HF I trial, which highlighted the potential of SCG in managing heart failure patients [14]. As the field evolves, the integration of FEM and digital twins based on SCG signals will play a crucial role in advancing our understanding of these signals under various anatomical, physiological, and pathological conditions. Despite the significant progress made, several limitations remain that need to be addressed to improve model accuracy and applicability. Key limitations include the following:

1. Simplified geometry: Although some of the prior studies have relied on 2D geometries to simplify SCG simulation, such an approach limits the ability to capture the inherently three‐dimensional propagation of cardiac‐induced vibrations. SCG signals arise in three directions (i.e., dorsoventral, right‐to‐left, and head‐to‐foot), and because the thorax is asymmetric (e.g., the heart sits to the left and has intricate anatomical structures), any 2D cross‐section inevitably misses vital geometric details. Moreover, SCG waveforms reflect subtle events (e.g., valve movements and chamber deformations) whose wave propagation cannot be fully represented in just 2D modelings. This often yields imprecise vibrational patterns and energy distributions on the chest surface. Consequently, 2D models cannot replicate the full SCG waveform, and transitioning to 3D geometries is strongly recommended for more accurate wave propagation modeling and closer alignment with clinical observations.
2. Incomplete organ modeling: There is a lack of comprehensive 3D models in the current literature that incorporate not only the heart but also surrounding organs and tissues. Such models are essential for accurately simulating the complex interactions within the thoracic cavity, including wave reflections and absorptions by neighboring structures. Future studies should aim to construct detailed 3D models that capture the full anatomical and mechanical context in which SCG signals propagate.
3. Material property realism: Another significant limitation is the assumption of simplified or idealized material properties for thoracic structures. Current studies often do not use material properties that fully reflect the heterogeneous, viscoelastic, and anisotropic nature of human tissue, which affects how cardiac vibrations propagate. Research focused on characterizing realistic, heterogeneous material properties and incorporating these into FEM models would enhance the models' predictive power.
4. Patient‐specific image‐based modeling for SCG variability: Developing image‐based, patient‐specific simulations can pave the way toward creating digital twin models that allow detailed investigation of SCG signals across different physiological and pathological conditions for the same individual. For instance, by first modeling a healthy subject's SCG signal, we could then simulate various conditions by altering the geometry (e.g., incorporating aortic coarctation, septal defects, or varying cardiac wall thickness), material properties (e.g., adjusting valve characteristics for valvular disease), and boundary conditions. This approach would enable predictions of how a subject's SCG signal might appear under specific disease states, providing critical insights into SCG signal variability both within and across individuals. By systematically simulating these changes, patient‐specific models could deepen our understanding of SCG signal variations, enhancing diagnostic and monitoring accuracy for diverse cardiac conditions.
5. Potential SCG biomarkers for disease detection: An important extension of FEM‐based SCG modeling is the identification of specific biomarkers related to diseases. For example, the amplitude, timing, and morphology of SCG signals can be affected by factors like the contractility of heart muscle [68]. For example, a study on 45 heart failure patients suggested that SCG waveform structure changes less in decompensated heart failure patients than in compensated ones after exercise, and therefore, such information can be utilized to track the clinical status of the patients [69]. Other studies suggested that a decrease in AO and AC amplitudes reflects a loss of mechanical strength in the heart, while shortened left ventricular ejection time and decreased pulse transit time are associated with reduced cardiac performance and increased arterial stiffness due to fluid shifts associated with the prolonged bed rest [70]. SCG signals have patterns that are associated with elevated peak systolic velocity, which is a key indicator of aortic valve stenosis [71]. By systematically introducing pathological geometries (for instance, septal defect) or modifying boundary conditions like reduced myocardial contractility, within numerical simulations, FEMs can predict how SCG features (i.e., potential biomarkers) such as signal amplitudes, fiducial point timings, and frequency contents might change. These modeled scenarios can help in developing disease‐specific SCG signals and in developing clinical strategies for diagnosing or monitoring heart diseases.
6. Including dynamic heart models in SCG signal modeling: While using medical imaging‐derived boundary conditions as discussed in this review can replicate SCG signals, a fully coupled electromechanical heart model, including myocardial activation, blood flow, and valve dynamics, is important for exploring how structural heart diseases, such as valve stenosis or myocardial infarction, alter SCG waveforms. Such integrated modeling would allow researchers to predict SCG changes when heart function (e.g., myocardial contractility or valve function) is compromised.

## 10 Conclusions

In conclusion, this review highlights the current advancements and limitations in FEM of seismocardiogram signals. FEM approaches provide valuable insights into the relationship between cardiac motion and chest surface vibrations, facilitating the non‐invasive assessment of cardiovascular function. However, limitations such as simplified 2D models, incomplete 3D anatomical representations, and inadequate material property characterizations underscore the need for further refinement. Addressing these challenges with advanced, patient‐specific models holds promise for improving diagnostic accuracy and personalized monitoring of cardiovascular health. Future efforts should focus on developing personalized, 3D FEM models that incorporate realistic anatomical and material properties, as well as adaptable digital twin models, to enhance the predictive power and clinical utility of SCG‐based assessments.

## Nomenclature

**Equation Terms**
x
: 3D position vector [m]
$f\left(t\right)$
: force [N]
$C\left(x\right)$
: elasticity tensor [Pa]
M
: mass matrix [kg]
C
: damping matrix [kg s^−1^]
K
: stiffness matrix [N m^−1^]
$q\left(x,t\right)$
: displacement vector [m]
$\dot{q}\left(x,t\right)$
: velocity vector [m/s].
$\ddot{q}\left(x,t\right)$
: acceleration vector [m/s^2^].
$\rho \left(x\right)$
: density [kg/m^3^]
σ
: stress tensor [Pa]
ϵ
: strain tensor
u
: prescribed displacement vector [m]
c
: wave propagation speed [m s^−1^]
E
: Young's modulus [Pa]
v
: Poisson's ratio
α,β
: Rayleigh damping coefficients

**Medical Terms**
SCG: seismocardiography
ECG: electrocardiography
FEM: finite element model(ing)
CT: computed tomography
AC: aortic valve closure
AO: aortic valve opening
MC: mitral valve closure
MO: mitral valve opening
CVD: cardiovascular disease

## Ethics Statement

The authors have nothing to report.

## Conflicts of Interest

The authors declare no conflicts of interest.

## Data Availability

Data sharing not applicable to this article as no datasets were generated or analysed during the current study.
